# Determination of Antibiotic Resistance Genes in the Interior Bay of Puno-Peru, Lake Titicaca

**DOI:** 10.1155/sci5/5571355

**Published:** 2025-03-21

**Authors:** Pompeyo Ferro, Jhordan Rossel, Ana Lucia Ferro-Gonzales, Eli Morales-Rojas, Euclides Ticona, Romel Guevara, Lizbeth Córdova

**Affiliations:** ^1^Faculty of Natural and Applied Sciences of the Universidad Nacional Intercultural Fabiola Salazar Leguia de Bagua, Jr. Ancash 520, Bagua 01721, Amazonas, Peru; ^2^Universidad Privada San Carlos, Ilave. Jr. Ilo 343 Ilave, Puno, Peru; ^3^Economic, Social and Strategic Development Research Group of the Universidad Nacional de Juliaca, Av. Nueva Zelandia 631, Puno 21101, Peru; ^4^Institute for Research in Information and Communication Technologies (IITIC) of the Universidad Nacional Toribio Rodríguez de Mendoza de Amazonas, Jr. Libertad No. 1300, Bagua, Amazonas, Peru

**Keywords:** antibiotic resistance genes (ARGs), Lake Titicaca, qPCR

## Abstract

Water can serve as a source of genetic resistance and act as an amplifier and/or reservoir for genes acquired by human pathogens, which can be released into the environment as pollutants. The interior bay of Puno, part of Lake Titicaca, is a popular tourist attraction, being an active component of the dynamics of the city of Puno. Therefore, the determination of the presence of antibiotic resistance genes (ARGs) in water samples from the interior bay of Puno of six collection points was the main objective of this research work. DNA extraction was conducted, followed by the identification and quantification of 16S rRNA and *Escherichia coli uidA* gene, two ARGs (*bla*_TEM_ and qacEΔ1), and class 1 integron-integrase gene (*intI1*) by means of quantitative PCR. The *intI1* and qacEΔ1 genes were detected throughout the interior bay of Puno; however, the abundance of the *bla*_TEM_ gene was comparatively lower. The uidA gene was reported only in some sampled points with < LOQ. These findings should raise concerns regarding the potential risk of their dissemination in Lake Titicaca and their impact on public health.

## 1. Introduction

Water is a fundamental element for human existence. It is a nonrenewable resource that is increasingly scarce, posing nowadays as one of the biggest problems in terms of its quality and management for human consumption. Every year, around two million people in the world, most of them children under five, die due to diseases of water origin, product of the consumption of contaminated water [1].

Tamames et al. [2] concluded that soil and fresh water, including aquifers, groundwater, lakes, rivers, and wastewater, are the natural habitats that host the largest and most diverse group of bacterial lineages. Similarly, the authors of [3] mentioned that there is still limited knowledge about the lineages harboring antibiotic resistance genes (ARGs) of clinical importance; therefore, drinking water obtained from natural sources has a high probability of containing bacteria (such as *proteobacteria* for example) and harbors ARGs.

Previous studies have suggested that bacteria present in natural habitats are potential sources of ARGs which, in some way, can be transmitted to human commensal and pathogenic bacteria; for example, indigenous soil microbiota has been identified as an important reservoir of resistance determinants that can be mobilized within the microbial community [4].

As a microbial habitat, water may serve as the origin of genetic resistance, be an amplifier and/or reservoir of genes acquired by human pathogens, and release them as contaminants in the environment [5]. Water can also act as a bioreactor, facilitating the exchange of resistance genes between pathogenic and nonpathogenic bacteria [6–9]. Moreover, water is one of the most important bacterial habitats on Earth and plays a significant role in the dissemination of microorganisms in nature, being also recognized as an important reservoir of antibiotic resistance [6, 9–11].

Antibiotics are not only selector agents for resistance mechanisms but also agents of acceleration of the evolution of resistance [12]. This is a consequence of the enormous plasticity observed in ARGs, allowing them to acquire new spectrum of action or enhanced capabilities beyond their original spectrum [13].

In the same trend, the authors of [3] indicated that despite intensive research in this area in recent years, it remains unclear under what circumstances bacteria in water serve as an important source of new resistance mechanisms or act as a vehicle or facilitator in the spread of antibiotic resistance; likewise, an unanswered question revolves around the potential relevance of antibiotic resistance in water to human health. Since resistance is harbored and transmitted by bacteria, gaining a deeper understanding of bacterial diversity and ecology can provide valuable insights into the spread of resistance. This approach is now possible because many studies from around the world in recent decades have been exploring bacterial diversity in water habitats.

Antibiotic resistance is increasingly becoming a problem that affects countries worldwide and has become the leading public health concern for some countries. Antibiotics are currently indispensable in the treatment of infectious diseases in both human and veterinary medicine [11] and are being addressed even under the One Health approach [14]. This issue is further aggravated by the mass dumping of antibiotics into surface, ground, and ocean waters, leading to a rapid spread of ARGs. While previous studies suggested that high levels of relevant ARGs resulted from the selective pressure caused by antibiotic contamination [15], recent research shows that the presence of resistance genes could be primarily due to fecal contamination, rather than solely selective pressure [16]. Attempts have been made for years to solve the problems of antibiotic resistance through continuous development new antibiotics, which resulted in the misuse and abuse of antibiotics worldwide [17]. Meanwhile, the threat of antibiotics resistance to public health and therefore to the One Health approach has not diminished; on the contrary, the number of deaths caused by resistance to antibiotics could reach 10 million per year by 2050 unless world leaders take appropriate measures [18–20]. The human population is still threatened by bacterial infections, without treatment in some cases, putting the One Health approach at risk; for example, the epidemics of *Streptococcus pneumoniae* and *Mycobacterium tuberculosis* [21] and the appearance of Gram-negative multiresistant bacilli [22] have already caused severe damage to human and animal health; therefore, the One Health approach is put at risk. At present, ARGs as contaminants are a concern due to their role in the propagation of resistance to antimicrobials, which are present in the environment [23], being a hot topic of research on both their transport and dissemination [24]. On the other hand, the use of fertilizers on agricultural land is a potential route for the transmission of antibiotic-resistant bacteria (ARB) from crops to animals and humans [14, 25]; additionally, rainfall can have a great impact on the abundance of ARGs [26]. Likewise, it has been reported that microorganisms are specific, which is closely related to the propagation of ARG, with different *intI1* genotypes inducing the expression of *sul1* and *ermF* genes in *Gammaproteobacteria* and *Bacteroidetes* [27]. The factor that most affects the transport and distribution of ARGs is the water environment. ARGs can diffuse through water bodies and can also be transferred between bacteria through horizontal gene transfer, contributing to their spread [28].

Lake Titicaca is one of the largest lakes in South America and extends along the border between Peru and Bolivia. The water from this lake is used for livestock, agriculture, recreation, and human consumption. Peruvian environmental quality standards are classified according to the use of the water [29]. The bay of Puno, which is located in the lake, has sediments with arsenic levels that exceed the maximum permitted limits according to regulation [30]. The problem increases even more due to the wastewater treatment plant (WWTP), which has a removal efficiency of 7.8% of total dissolved solids [31], which discharges its effluents into the bay; this is evidenced by studies carried out that show that physical–chemical parameters in areas close to the WWTP effluents do not comply with Peruvian directive [32, 33]. However, research about bacterial pollution and ARGs is scarce and null, respectively.

For everything written in the lines above, it is extremely important to screen for the presence of ARGs in water, particularly if it is used for human consumption. This research was carried out in order to detect ARGs and indicators of contamination that determine the quality of the water, in the interior bay of the city of Puno, being part of the highest lake in the world, Lake Titicaca, considering lakes provide an important environment to study antibiotics and ARG transport with a wide range of niches including bacterial communities, aquatic plants, and animals [11, 34, 35].

## 2. Materials and Methods

### 2.1. Delimitation of the Study and Sampling

The interior bay of Puno, or the interior bay of Titicaca, is a small section of Lake Titicaca, located in front of the city of Puno, Puno Region, and in the southeast of Peru. It extends between the promontories of Chulluni to the north and Chimú to the south, covering a total area of 15.91 km^2^. Inside the bay, a large number of reeds grow that restrict the calm flow of water between the interior bay and Lake Titicaca. This situation facilitates the accumulation of pollutants and sediments. Furthermore, Lake Titicaca is an environment that can host and disseminate antibiotic resistance factors, as it receives poorly treated wastewater, waste from inhabitants [36], and stormwater runoff [37], which worsens this situation, considering also that the lake is visited by local and foreign tourists.

**Figure 1:**
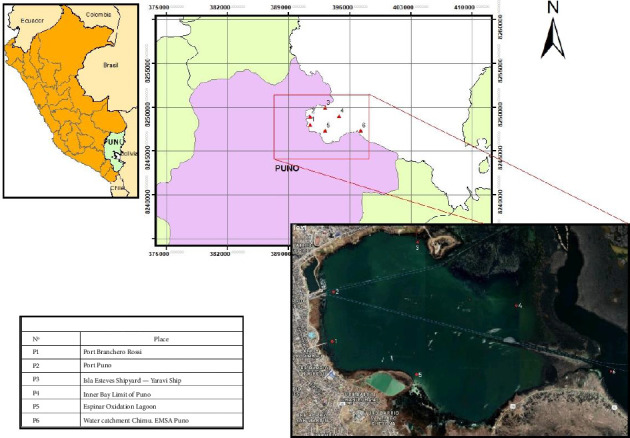
Geographical location of the sampling points in the interior bay of Puno.

Sampling was carried out in April 2019 (three repetitions on different dates), collecting samples (duly georeferenced) from six points in the interior bay (Figure 1). The sampling points were chosen according to nonprobabilistic statistics, in order to identify variability and greater precision:

a. Point close to the water catchment for human consumption in the city of Puno (P6).
b. Point located in the port where there is a lot of movement of local and foreign people (P2).
c. Point located within the inner bay of the lake (P4).
d. Point close to the wastewater oxidation lagoon (P5).
e. Point located on Esteves Island where there is a tourist hotel (P3).
f. Point located in a secondary port that exists in Puno (P1).

In addition, the research was carried out, under the criteria established by the Ministry of Health [38, 39] maintaining the following considerations: 3.5 L of water was collected for sample collection, using sterile PET bottles of 3.5 L capacity, one per sampling point; the samples are from surface water (interior bay of Puno), being collected within 15 cm of depth, following the protocol of rinsing each bottle 3 times with the water sampled at each programmed point, later being sealed and placed in a cooler with freezing gel, to be preserved and avoid the incidence of solar radiation until they are transported to the laboratory (within 4 h of taking the samples). One liter of the sampled water was filtered using cellulose ester filters with a porosity of 0.45 μm and 47 mm in diameter. This process was carried out in the laboratories of the Private University of San Carlos de Puno.

### 2.2. Extraction of DNA

The PowerWater DNA Isolation Kit (MO BIO Laboratories, Inc., 2746 Loker Ave West, Carlsbad, CA 92010) was used following the recommended protocol by the manufacturer. In addition, quality control measures were applied during extraction to ensure the integrity of the DNA, working in a safety cabinet. The extracted DNA has been conditioned in a styrofoam transport box (from Puno, Perú, to Porto, Portugal), under freezing conditions in nitrogen gas. DNA concentrations were relatively low, between 2.99 ng/μL and 18.05 ng/μL.

### 2.3. Quantitative PCR (qPCR)

It has been carried out by means of qPCR, in the facilities of the Superior School of Biotechnology of the Portuguese Catholic University in Porto, Portugal; first it was carried out for 16S rRNA and later to identify ARGs: *uidA*, *bla*_TEM_, *qacEΔ1*, and *intl1*, under the following considerations.

Targets for detection and quantification by qPCR (all P1–P6 sampling points) were 16S rRNA (total bacteria load) and *Escherichia coli uidA* genes, two ARGs (*bla*_TEM_ and *qacEΔ1*), and class 1 integron-integrase gene (*intI1*).

SYBR Green qPCR assays were conducted in a StepOnePlus Real-Time PCR System (Life Technologies, USA), and by using the standard curve method described in [40] for absolute gene quantification. qPCR assays for genes 16S RNA, *bla*_TEM_, and *intI1* were performed in all DNA samples (total of 18 DNA extracts, 3 DNA extracts per sampling point), but for the genes *uidA* and *qacEΔ1* we selected the DNA extract with the highest concentration per sampling point (total of 6 samples for qPCR). Information on gene-specific primer sequences, cycling conditions, and DNA samples used to construct the standard curves per gene (including the limit of quantification (LOQ)) is provided in Table 1. Quality criteria described in [45] were further applied, and data were expressed as log_10_ of the ratio of gene copy number per mL of water (log_10_ (gene copy number/mL of water). This was chosen in order to normalize the distribution of gene abundance values and avoid the dataset being too skewed if raw counts are used. Also because the log_10_ transformation reduces this asymmetry when transforming the data, since in these proportions, it is easier to compare the differences in gene abundance at the sampling points.

The rationale for the selection of the 16S rRNA genes, uidA, blaTEM, qacEΔ1, and intI1 in our study is based on their critical roles in assessing microbial contamination and antibiotic resistance. (a) 16S rRNA: It serves as a universal marker for bacterial identification, allowing us to estimate the total bacterial load in water samples. (b) uidA: It indicates fecal contamination, as it is specific to *Escherichia coli*, helping to assess the risk of waterborne pathogens. (c) blaTEM: It encodes a beta-lactamase enzyme, signifying the presence of antibiotic resistance, which is crucial for public health. (d) qacEΔ1: It confers resistance to biocides, indicating possible resistance mechanisms in bacteria exposed to disinfectants. (e) intI1: It is associated with horizontal transfer of antibiotic-resistant genes, highlighting the spread of resistance in microbial communities. By analyzing these genes, we provide a comprehensive view of microbial contamination and antibiotic resistance in the inner bay of Puno, providing valuable insights into public health risks.

The data analysis focused on a direct visual comparison of data between sampling points, with the aim of identifying the presence and variability of genes, which will serve as a basis for future studies.

## 3. Results

**Figure 2:**
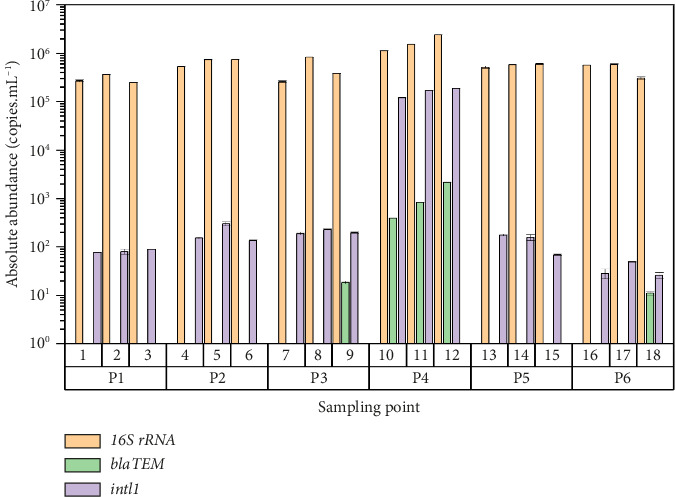
The absolute abundance of *bla*_*TEM*_ and *intl1* genes in water in bay of Puno.

The presence of 16S rRNA was absolute, with an abundance ranging from 2.48 × 10^5^ to 2.4 × 10^6^ copies.mL^−1^, as can be seen in Figure 2. The *intl1* gene was detected in all samples and sampling points, reporting in the inner bay limit of Puno the greatest abundance ranging from 2.57 × 10^1^ to 1.86 × 10^5^. While the *bla*_TEM_ gene was only detected in three sampled points (P3, P4, and P6), of these points, only sampling point P4 showed presence of the *bla*_TEM_ gene in its three samples.

Regarding to *uidA* and *qacEΔ1* genes were only amplified one sample per sampling point, of which *uidA* gene was detected in all sampling points (9.31–2.26 × 10^5^ copies.mL^−1^) while *qacEΔ1* gene only was detected in P4 (6.22 × 10^1^ copies.mL^−1^) as can be seen inFigure 3,comparatively, the 16S rRNA and *intl1* genes were reported with absolute presence with respect to the rest of genes.

## 4. Discussion

An absolute abundance of the *int1* gene is appreciated, similar to what was reported in [27, 46] in all the points sampled in the interior bay of Puno; this gene expresses resistance to antibiotics, disinfectants, and heavy metals [47]; furthermore is strongly correlated with *sul1, sul2, ErmB y qnrS* [48]; consequently, its presence in the water of the interior bay of Puno, the main component of Lake Titicaca, reflects a high degree of contamination to ARG, since it was practically detected in the entire interior bay (Figures 1 and 2), which makes a lot of sense since ARGs are correlated with metal resistance genes (MRGs) [49], which in turn have been reported in high concentrations in sediment from the bay of Puno [30]. This can be considered serious because the interior bay of Puno receives permanent visits throughout the year from national and international tourists, being a very important tourist attraction in the Puno Region; in addition, on the other hand, its presence in the sampled point is more worrisome, close to the collection of water for human consumption, a concern also shared in similar publications regarding ARG contamination [50]. This is very likely due to the fact that the feces of humans, birds, cattle, and fish [28] are washed away by the rain runoff toward the interior bay of Puno, also due to the migration of physical factors, like the flow of water and winds. In addition to aggravating this because the discharges from the oxidation lagoon of the city of Puno, is to the interior bay of Puno as shown in Figure 2. On the other hand, some studies indicate that bird feces contain a large amount of ARG [51, 52]. Thus, migratory birds that pass through the bay and Lake Titicaca can spread ARG. It is important to point out that this oxidation lagoon no longer fulfills any oxidation function, evacuating the wastewater that it houses directly to the interior bay of Puno, currently without any treatment, and this is one of the sources of the spread of antibiotic resistance [11, 26, 35, 53–56], considering also that the resistance genes can be explained to a great extent by the contamination of feces [57]. The presence of this *int1* gene, having the capacity to incorporate various xenogenetic elements [47], generates a legitimate concern which should be shared by the Puno authorities to avoid contamination of the bay by sewage from the Puno city, as they may be playing a role in the dispersal and dissemination of ARGs in the lake [27, 58], since its abundance could also be explained by levels of fecal contamination [57].

Beta-lactams are one of the antibiotics that are widely used in both human and veterinary medicine, precisely because it does not generate too many side effects; therefore, its presence in the environment is very persistent. The *bla*_TEM_ gene is one of those responsible for the expression of resistance to beta-lactams, due to its location in a plasmid attached to a transposon, it allows transfer between different species [28] and it is clinically relevant already reported in water samples [59]. In the interior bay of Puno, the *bla*_TEM_ gene has only been reported in three sample points located on the border with the rest of Lake Titicaca, since beta-lactam antibiotics in the environment are sensitive to pH and temperature and are easily degraded [60], making the abundance of the *bla*_TEM_ gene in the environment lower than expected [28].

Regarding the *qacEΔ1* gene that confers resistance to biocides, it has been reported only once out of the three times sampled, but at each sampled point, it has been found throughout the bay interior of Puno, similar to what was mentioned in a review article on ARGs [35, 61, 62]; this gen can be transferred horizontally [62].

Due to the fact that AGRs have been reported in the interior bay of Puno, the company that provides the water service for human consumption, in this case EMSAPUNO (company that provides the drinking water service in the city of Puno), should explore the possibility of implementing a surveillance program in the water for human consumption in the city of Puno, of antimicrobial resistance factors, and thus contribute to reducing the negative impacts of resistance to antibiotics under the framework of One Health [56]; although it is important to recognize that in the world, WWTPs are not designed to eliminate ARG [54], and obviously Puno does not escape this, in addition, EMSAPUNO must take into account that the water disinfection process, far from helping to eliminate ARG, promotes the horizontal genetic transfer of ARB [63], therefore, it denotes the need to implement ultraviolet disinfection methods, for example, to reduce the threat of dissemination [27].

## 5. Conclusions

The *int1* and *qacEΔ1* genes were reported in all the sampling points of the interior bay of Puno, which undoubtedly generates great concern about the risk of dissemination, especially at the sampling point close to the collection of water for human consumption of the city of Puno.

Comparatively, the abundance of the *bla*_TEM_ gene is low in the interior bay of Puno, but its presence should also be considered with caution.

The drinking water company of the city of Puno, EMSAPUNO, should consider a surveillance program for antimicrobial resistance factors in drinking water.

## Figures and Tables

**Figure 3:**
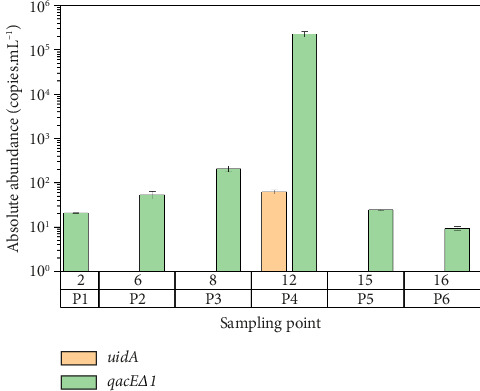
The absolute abundance of *uidA* and *qacEΔ1* genes in water in bay of Puno.

**Table 1:** Quantitative PCR conditions used in the present study for absolute gene quantification in water samples.

Target (gene or class)	Primer sequences (5′ to 3′)			Reference DNA	LOQ	Cycling conditions^†^
16S rRNA	1114	CGGCAACGAGCGCAACCC	[40]	*E. coli* ATCC 25992	1033	Initial denaturation step at 95°C for 10 min;
	1275	CCATTGTAGCACGTGTGTAGCC				15 s at 95°C, 20 s at 55°C, and 10 s at 72°C (35 cycles)^1^

*uidA*	uidA_F	CAACGAACTGAACTGGCAGA	[41]	Clone *uidA* (*E. coli* A2FCC14)	16	Initial denaturation step at 95°C for 10 min;
	uidA_R	CATTACGCTGCGATGGAT				15 s at 95°C, and 1 min at 60°C (40 cycles)^2^

*bla*_TEM_	blaTEM-F	TTCCTGTTTTTGCTCACCCAG	[42]	pNORM1	70	Initial denaturation step at 95°C for 10 min;
	blaTEM-R	CTCAAGGATCTTACCGCTGTTG				15 s at 95°C, and 1 min at 60°C (40 cycles)^2^

*qacEΔ1*	qacEΔ1-02F	CCCCTTCCGCCGTTGT	[43]	Clone *qacEΔ1* (*E. coli* A4FC16)	18	Initial denaturation step at 95°C for 5 min;
	qacEΔ1-02R	CGACCAGACTGCATAAGCAACA				10 s at 95°C, and 30 s at 60°C (35 cycles)^2^

*intI1*	intI1-LC1	GCCTTGATGTTACCCGAGAG	[44]	pNORM1	70	Initial denaturation step at 95°C for 10 min;
	intI1-LC5	GATCGGTCGAATGCGTGT				15 s at 95°C and 1 min at 60 °C (40 cycles)^3^

*Note:* Limit of quantification. LOQ (copy number/2 μL target gene).

^†^StepOnePlus real-time PCR system (Life Technologies, USA).

^1^KAPA SYBR FAST qPCR kit. ABI prism. 200 nM of primer in a reaction volume of 20 μL.

^2^SYBR select master Mix. applied biosystems. 200 nM of primer in a reaction volume of 20 μL.

^3^Power SYBR green PCR master Mix. applied biosystems. 200 nM of primer in a reaction volume of 20 μL.

## Data Availability

The data used to support the findings of this study are available from the corresponding author upon request.
